# Potential of Subterranean Microbes: High‐Throughput Screening for Industrially Relevant Enzymatic Activities in Dinaric Caves

**DOI:** 10.1002/mbo3.70146

**Published:** 2025-11-05

**Authors:** Justinas Babinskas, Jokūbas Krutkevičius, Lada Lukić Bilela, Renata Bešta‐Gajević, Inga Matijošytė

**Affiliations:** ^1^ Life Sciences Center, Institute of Biotechnology, Sector of Applied Biocatalysis Vilnius University Vilnius LT Lithuania; ^2^ Department of Biology, Faculty of Science University of Sarajevo Sarajevo Bosnia and Herzegovina; ^3^ Biospeleological Society in Bosnia and Herzegovina (BIOSPELD) Sarajevo Bosnia and Herzegovina

## Abstract

Enzymes derived from extremophiles, or extremozymes, possess unique properties that enable them to function under extreme environmental conditions. Microbial communities in subterranean ecosystems have evolved specialized metabolic pathways to survive, leading to the discovery of bioactive molecules with diverse biotechnological and industrial applications as well as the development of sustainable methods for habitat restoration. This study aimed to identify cultivable microorganisms producing industrially relevant enzymes, such as laccases, proteases, and urethanases, from extremophiles in the Dinaric Karst subterranean ecosystems, which are known as biodiversity hotspot. A total of 40 samples were collected from six caves and an abandoned railway tunnel, now a key roost for a large *Myotis myotis* maternity colony. Cave samples were taken from the entrance, twilight, and dark zones, including soil, sediments, moonmilk, mineral deposits, bedrock deposits, insect remains, entomophagous fungi, wall biofilm, and guano from various bat species. Following microbial cultivation, 207 colonies were screened for enzymatic activity using substrate‐specific assays. Functional analysis identified one microorganism exhibiting strong laccase activity, seven capable of degrading polyurethane, and numerous protease‐producing colonies. Notably, this study constitutes the inaugural report on discovering polyurethane‐degrading microorganisms in karst caves. Molecular identification revealed microbial genera, including *Bacillus*, *Pseudomonas*, *Serratia*, *Paenibacillus*, and *Priestia*. These findings underscore the biotechnological potential of subterranean extremophiles and highlight the importance of conserving these ecosystems. Further characterization of these enzymes may drive advancements in environmental remediation, waste recycling, and sustainable industrial processes.

## Graphical abstract

Microbial communities from seven Dinaric cave ecosystems were surveyed for their laccase, urethanase, and protease enzymatic activities. The findings suggested potential applications in bioremediation, the biodegradation of synthetic polymers, and industrial biotechnology, highlighting the importance of biodiversity conservation.

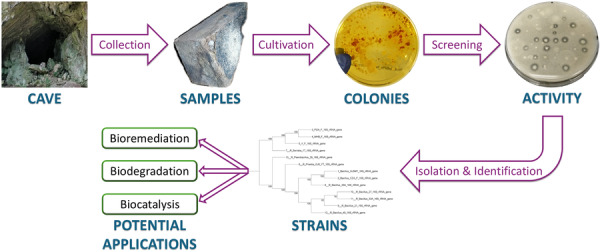

## 1 Introduction

Enzymes are crucial for various industrial applications, particularly in biocatalysis, where they can perform reactions more efficiently and sustainably than traditional chemical methods. Biocatalysis plays a pivotal role in advancing a sustainable, circular, bio‐based economy, with significant progress in biotechnology over the past decades (Mesbah 2022). From an industrial perspective, an ideal biocatalyst is stable and active under processing conditions, has exposed substrate specificity, regioselectivity, and enantioselectivity, operates efficiently to produce the desired product promptly, and minimizes by‐products and product inhibition (Buller et al. 2023). Most commercially available enzymes are derived from mesophilic organisms, whose limited stability restricts their applicability in various processes (Cabrera and Blamey 2018). In contrast, extremophilic microorganisms, which thrive under such conditions, produce resilient enzymes, known as extremozymes, with superior stability and activity in unconventional environments. Extremozymes exhibit unique adaptations that enable them to function optimally in harsh environments (Littlechild 2015), surpassing their mesophilic counterparts in applications under extreme conditions (Elleuche et al. 2014). They are categorized based on their natural habitats—thermophilic, psychrophilic, halophilic, acidophilic, and alkaliphilic enzymes. Thermophilic enzymes are active at temperatures ranging from 50°C to 125°C; these enzymes benefit from structural features such as disulfide bridges that enhance thermal stability. They are particularly useful in processes that require high temperatures to reduce contamination risks and increase reaction rates. Psychrophilic enzymes operate effectively at low temperatures, −20°C to 10°C, lowering the activation energy required for reactions. Their flexibility allows them to maintain catalytic activity despite the cold. Halophilic enzymes are found in high‐salinity environments at sodium concentrations greater than 1.5 M. Their adaptations include an abundance of acidic amino acids that enhance solubility and flexibility in ionic conditions. Acidophilic and alkaliphilic enzymes are adapted to extreme pH levels, with acidophiles thriving in low pH environments and alkaliphiles in high pH conditions. Their stability is often linked to specific structural features that protect them from denaturation.

Nevertheless, the availability of extremozymes remains limited due to challenges in cultivating extremophiles in laboratory environments to meet the growing industrial demand, as well as their accessibility in extreme habitats. Additionally, the lack of effective methodologies for their functional analysis further complicates the process (Mesbah 2022). While culture‐independent methods such as sequence‐based metagenomics (SBM) and single amplified genomes (SAG) can facilitate the identification of genes that may encode enzymes of interest (Sysoev et al. 2021), this study exclusively employs the functional culture‐dependent approach. This method allows the discovery of natural extremozymes capable of catalyzing specific reactions under targeted conditions. In this approach, extremophiles are cultured and isolated under selective pressures and then screened based on their enzymatic activity to identify the most promising candidates for further research (Espina et al. 2022). Using this approach, the present study aims to broaden the scope of extremophile research by identifying enzymes of industrial interest within subterranean microbial communities from Dinaric caves. Caves, mostly dark zones, are considered extreme environments that offer unique niches for highly specialized microorganisms (Afzal et al. 2021). In particular, diverse psychrophilic (cold‐loving) and psychrotolerant (cold‐adapted) microorganisms produce enzymes that remain active at low temperatures, which is essential for their survival in such harsh conditions (Tomova et al. 2013). Despite limitations in energy input, subterranean ecosystems are usually inhabited by diverse microbial communities (Barton and Jurado 2007; Engel 2015). Characterized by the absence of photosynthetic activity, high relative humidity, low rates of evaporation, and stable temperature, low‐energy underground environments are uniquely suited for examining the minimum energetic requirements and adaptations for chemolithotrophic life (Jones et al. 2018). Microbes that inhabit these ecosystems exhibit remarkable survival strategies due to their ability to modify and adapt their metabolic pathways in response to harsh and resource‐limited environments that allow them to exploit available resources efficiently (Kosznik‐Kwaśnicka et al. 2022; Khatri et al. 2024; Salazar‐Hamm et al. 2025). Further research and elucidation of their highly specialized metabolic pathways and biochemical processes may lead to the discovery of new bioactive molecules with various biotechnological and industrial applications and eco‐friendly bioremediation techniques.

The Western Balkan's Dinaric Karst is widely recognized as one of the global hotspots of subterranean biodiversity (C. Culver et al. 2006; Zagmajster et al. 2018), including two cave systems with the highest biodiversity of subterranean fauna in the world: the Vjetrenica Cave System in Bosnia and Herzegovina (Delić et al. 2023) and the Postojna‐Planina Cave System (PPCS) in Slovenia (Zagmajster et al. 2021). Dinaric subterranean ecosystems are also characterized by distinct biodiversity of microbial communities (Mulec 2008; Pašić et al. 2010; Kostanjšek et al. 2013; Mulec and Summers Engel 2019), which hold significant potential for biotechnological applications (Kosznik‐Kwaśnicka et al. 2022), making them a promising area for further research (Ghosh et al. 2017; Martin‐Pozas et al. 2020).

The study of laccase, protease and urethanase activities derived from extremophiles represents a promising area of research with significant implications for environmental and industrial biotechnology. Laccases are versatile enzymes with considerable potential for application in several industrial contexts, particularly in the remediation of contaminated sites, the processing of textiles, the production of foodstuffs, and the remediation of the environment. Their capacity to oxidize a diverse array of substrates renders them a valuable tool in the remediation of environmental pollutants and optimizing industrial processes (Brugnari et al. 2021; Leynaud‐Kieffer et al. 2022). Proteases are enzymes that catalyze the breakdown of proteins into smaller peptides or amino acids. They have various industrial applications, including the food and feed, detergent and leather industries, pharmaceuticals, and bioremediation (N. Jisha et al. 2013; Aruna et al. 2023; Song et al. 2023). Urethanases have yet to be extensively investigated; they catalyze the hydrolysis of urethane compounds, making them of significant interest in recycling polyurethane. As environmental concerns intensify, the role of urethanases in waste reduction and material innovation is expected to expand further (Raczyńska et al. 2024). Continued exploration and characterization of these enzymes will likely lead to new biotechnological advancements that leverage their capabilities, adopted from extreme environmental conditions. Moreover, these discoveries can significantly improve the protection of subterranean habitats and the conservation of their biodiversity by finding new bioactive molecules and environmentally acceptable solutions as a result of multidisciplinary research.

## 2 Materials and Methods

### 2.1 Sampling Sites and the Sampling Methodology

**Figure 1:**
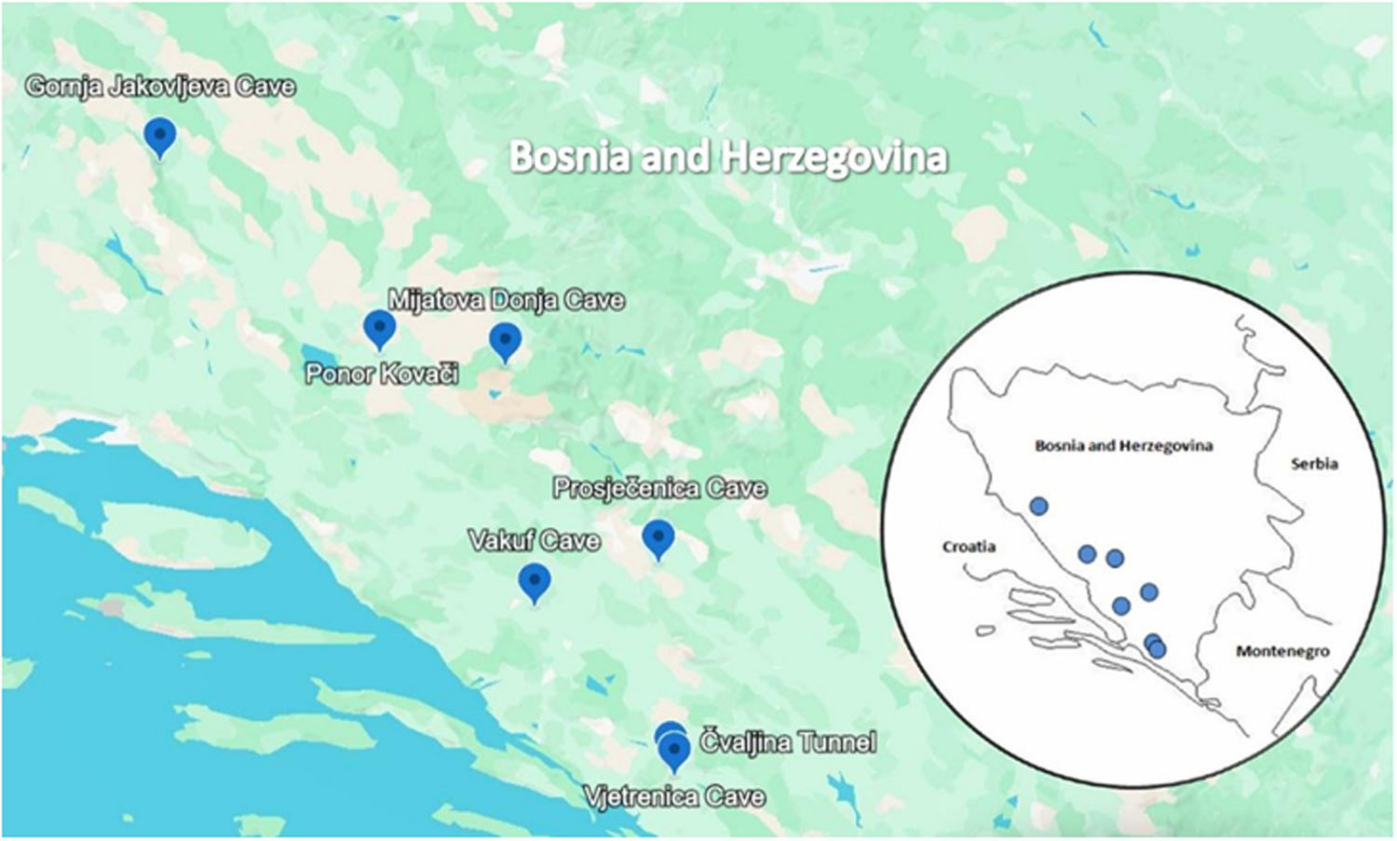
The map of the sampling sites in Bosnia and Herzegovina.

The samples were collected during the International speleological and speleodiving expeditions “Ponor Kovači—Izvor Ričine” (Tomislavgrad) in the period from August to September 2021 and 2022, and the Balkan Cave Summit (Mostar) 2022, at seven localities in Bosnia and Herzegovina, including six caves, and one abandoned, above‐ground railway tunnel, whose elevations vary from 57 to 1310 m (Figure 1; Table 1). Vjetrenica Cave (Zavala, Popovo polje) is one of the world's most important hotspots of subterranean fauna biodiversity, inscribed on the UNESCO List of Natural Heritage in 2024. It is one of the longest caves in Bosnia and Herzegovina, with a length of 7.324 m (Ozimec and Baković 2021) and forms part of the Trebišnjica river system, once the longest underground river in Europe. This cave is part of a complex Vjetrenica Cave System that, along with Vjetrenica, includes the Bjelušica Cave and the Lukavac spring, in which more than 230 taxa have been recorded so far, with a total of 93 troglobiotic, that is, obligate subterranean aquatic (48) and terrestrial (45) taxa (Delić et al. 2023). Furthermore, some sampling sites are known as the important bat habitats within the Natura (2000) (N2K) sites. Thus, the tunnel in village Čvaljina (Popovo polje), about 300 m long, is the most roost on the broader area, sheltering a large maternity colony of *Myotis myotis*, with the possible presence of the *M. blythii*, numbering up to 10,000 (females with young) (Mazija and Rnjak 2016). More than 10 species of bats were recorded in Prosječenica cave (Podveležje, Velež), (Rnjak et al. 2017), and the only so far known maternity colony of *Rhinolophus blasii* on the Dinarid mainland was also found (Presetnik et al. 2014). In Vakuf Cave (Gornji Studenci), about 200 meters long, a maternity colony of *Myotis myotis/blythii* numbering about 500 specimens (Mulaomerović 2013) and *Myotis capaccini* was also found (Hodžić 2015).

**Table 1:** Sampling data of microbial communities in selected sampling sites.

	Gornja Jakovljeva Cave	Donja Mijatova Cave	Ponor Kovači	Prosječenica Cave	Vakuf Cave	Čvaljina Tunnel	Vjetrenica Cave
Locality	Dinara Mt.	Vran Mt.	Grabovica Plateau	Podveležje	Gornja Studenčica	Čvaljina, Popovo polje	Zavala, Popovo polje
Latitude	44.050578	43.653268	43.676821	43.26569	43.18007	42.86828	42.504521
Longtitude	16.582166	17.526883	17.187070	17.9402	17.60697	17.9719	17.59168
Air temperature	7.9°C	7.9°C	9.6°C	19.9°C	17.1°C	18.2°C	11.3°C
Water temperature	7.4°C	7.7°C	9.2°C	—	—	—	11.1°C
Bedrock temperature	7.5°C	7.5°C	9.2°C	—	—	—	11.2°C
Relative humidity	100%	100%	99%	85%	97%	—	100%
Altitude (m)	860	1310	868	660	57	307	260
Date of sampling	2.09.2022	24.08.2022	01.09.2022	30.08.2022	29.08.2022	31.08.2022	31.08.2022
Sampling legality	L.L.B; J.B.	L.L.B; J.B.	L.L.B; J.B.	L.L.B; J.B.	L.L.B; J.B.	L.L.B; J.B.	L.L.B; J.B.

The samples were collected from the cave entrance zone, the twilight zone, and the transition zone to the dark zone (Ghosh et al. 2017) using sterile forceps and transferred into sterile falcon tubes, transported by portable coolers to the laboratory and kept at +4 C until analysis. Bat guano deposits were collected with disposable plastic spoons or disposable wooden sticks, with possible removal of soil and sand. The sampling was carried out in compliance with safety regulations and wearing protective equipment; the samples were stored in marked plastic bags and then packed in jars for safe transport (Dimkić et al. 2021). Physical parameters were measured using a combined thermo‐hygro‐anemometer Kestrel 4000 Series Pocket Weather Meter and 4‐in‐1 environmental meter (luxmeter, sound level meter, thermometer, hygrometer) Voltcraft.

### 2.2 Composition of Agar‐Media Plates for Cultivation

*Modified Mueller‐Hinton (mMH) agar*. 2 g of beef extract, 5 g of yeast extract, 17.5 g of soybean peptone, 1.5 g of glucose, and 15 g of agar were suspended in 1 L of dH_2_O and autoclaved at 121°C for 20 min.

*Yeast extract‐peptone‐glycerol (YEPG) agar*. 10 g of yeast extract, 20 g of soybean peptone, 20 g of glycerol, and 15 g of agar were suspended in 1 L of dH_2_O and autoclaved at 121°C for 20 min.

*Ferbamine stock solution* was prepared as described in Babinskas et al. (2024). In short, 7.53 g of *N,N*‐dimethyl‐*p*‐phenylenediamine dihydrochloride was dissolved in 100 ml of dH_2_O. In small portions, 3.816 g of Na_2_CO_3_ was added, and the solution was cooled down to 0°C–5°C in an ice bath. (NH_4_)_2_S_2_O_8_ solution, made of 8.2 g of ammonium persulfate and 40 mL of dH_2_O, was cooled down to 0°C–5°C in an ice bath and added dropwise. After 3 h, the reaction mixture was warmed up to 20°C. Black precipitate was collected using filtration, washed with 4 × 100 mL of dH_2_O and dried over P_2_O_5_. A slurry of 150 mg of precipitate and 150 mL of ethyl acetate was refluxed for 1 h, filtered, washed with 4 × 50 mL of ethyl acetate, and dried in open air. Then, 50 mg of the refluxed precipitate was further refluxed in 100 mL of 5% (v/v) methanol‐water solution for 4 h. Insoluble residues were removed using filtration, and the Ferbamine was collected by evaporating the filtrate at 55°C under reduced pressure. Synthesis yielded 45 mg of Ferbamine dissolved in 1 L of dH_2_O to produce a 45 µg/mL Ferbamine stock solution.

*Ferbamine‐mMH agar*. Stock solution of mMH agar was prepared by suspending 2 g of beef extract, 5 g of yeast extract, 17.5 g of soybean peptone, 1.5 g of glucose, and 15 g of agar in 167 mL of dH_2_O and autoclaving it at 121°C for 20 min. 833 mL of Ferbamine stock solution was combined with the mMH stock and mixed at 70°C–80°C until the media became homogeneous.

*Ferbamine‐YEPG agar*. YEPG agar stock was prepared by suspending 10 g of yeast extract, 20 g of soybean peptone, 20 g of glycerol and 15 g of agar in 167 mL of dH_2_O and autoclaving it at 121°C for 20 min. 833 mL of Ferbamine stock solution was combined with the YEPG stock and mixed at 70°C–80°C until the media became homogeneous.

*Ferbamine‐potato‐dextrose agar*. Potato‐dextrose agar (PDA) stock solution was prepared by suspending 24 g of potato‐dextrose broth and 15 g of agar in 167 mL of dH_2_O and autoclaving it at 121°C for 20 min. 833 mL of Ferbamine stock solution was combined with the PDA stock and mixed at 70°C–80°C until the media became homogeneous.

*Ferbamine‐Luria‐Bertani agar*. Luria‐Bertani (LB) agar stock solution was prepared by suspending 25 g of LB broth (Miller's modification) and 15 g of agar in 167 mL of dH_2_O and autoclaving it at 121°C for 20 min. 833 mL of Ferbamine stock solution was combined with the LB stock and mixed at 70°C–80°C until the media became homogeneous.

*Ferbamine‐Czapek agar*. Czapek agar stock solution was prepared by suspending 49 g of Czapek‐agar in 167 mL of dH_2_O and autoclaving it at 121°C for 20 min. 833 mL of Ferbamine stock solution was combined with the Czapek stock and mixed at 70°C–80°C until the media became homogeneous.

*Minimal salt media (MSM) agar*. MSM stock solution was prepared by suspending 7 g K_2_HPO_4_, 2 g KH_2_PO_4_, 0.1 g MgSO_4_*7H_2_O, 0.001 g ZnSO_4_*7H_2_O, 0.0001 g CuSO_4_*5H_2_O, 0.0015 g MnCl_2_*4H_2_O, 0.01 g FeSO_4_*7H_2_O, in 100 mL of dH_2_O, and filter sterilized with Millipore 0.45 µm pore size PVDF membrane syringe filter (Merck, Germany). Agar stock solution was prepared by suspending 15 g of agar in 800 mL of dH_2_O and autoclaving it at 121°C for 20 min. MSM agar was prepared by mixing the MSM stock solution with all of the agar stock solution, and sterile dH_2_O was added to reach the final total volume of 1 L. MSM stock solution with additional carbon and nitrogen sources (MSM + N + C) was prepared by adding 1 g (NH_4_)_2_SO_4_ and 4.28 g monosodium citrate to the MSM stock solution. MSM stock solution with an additional nitrogen source (MSM + N) was prepared by adding 1 g (NH_4_)_2_SO_4_ to the MSM stock solution.

*MSM‐Polyurethane (PU) agar*. MSM‐PU agar was prepared by enriching MSM agar solution with polyurethane Impranil solution to a final concentration of 0.3% (v/v). Polyether (PE) Impranil DAA (Covestro, Germany) and polyester (PS) Impranil DLN‐SD (Covestro, Germany) polyurethane solutions were used to make MSM‐PE and MSM‐PS agar, respectively. MSM stock solution with additional carbon and nitrogen sources (MSM + N + C) and MSM stock solution with additional nitrogen source (MSM + N) were used to enrich MSM‐PU media. MSM‐PU agar composition was based on (Peng et al. 2014).

*Skim‐milk (SM) agar*: 28 g of SM powder, 5 g of tryptone, 2.5 g of yeast extract, 1 g of dextrose and 15 g of agar were suspended in 1 L of dH_2_O and autoclaved at 121°C for 20 min.

### 2.3 Cultivation of Microorganisms

Culturing microorganisms from collected samples was performed by placing a piece of the sample not larger than 1 cm × 1 cm × 1 cm in a Petri dish, suspending it in 5 mL of 0.9% NaCl solution, and streaking the suspension on the agar plate. For each sample, microorganism cultivation was performed on mMH at temperatures of 20°C and 37°C and on YEPG plates at temperatures of 20°C and 30°C. Plates were incubated until microbial colonies formed, but not for more than 168 h. Plates containing grown colonies were stored at 4°C until further use.

### 2.4 Screening for Enzymatic Activities

Every colony displaying distinct morphology traits (size, shape, color, texture and pigmentation) was carefully transferred with sterile toothpicks to the corresponding screening plate.

*Laccase activity* screening was conducted using media supplemented with Ferbamine at the same temperature as cultivation for the appropriate colonies: 20°C, 30°C or 37°C. The screening plates were examined every 24 h, up to 168 h.

*Polyurethane degradation assay* used MSM‐PU agar media. The media variants used were as follows: MSM‐PE (MSM media supplemented with polyether PU), MSM‐PS (MSM media supplemented with polyester PU), MSM + N‐PE (MSM media supplemented with polyether PU and nitrogen source), MSM + N‐PS (MSM media supplemented with polyester PU and nitrogen source), MSM + N + C‐PE (MSM media supplemented with polyether PU, nitrogen and carbon sources), MSM + N + C‐PS (MSM media supplemented with polyester PU, nitrogen and carbon sources). The activity screening was conducted at the same temperature as cultivation for the appropriate colonies—20°C, 30°C, or 37°C. The screening plates were examined every 24 h, up to 168 h. The formation of clearing zones around microorganisms was considered a positive result.

*Protease activity* was identified using SM‐agar plates. The plate incubations were conducted for 48 h and at the same temperature as the cultivation for the appropriate colonies—20°C, 30°C, or 37°C.

### 2.5 Identification of Microorganisms

The extraction of the microorganism DNA was carried out using the Quick‐DNA Fungal/Bacterial Miniprep Kit (Cat No. D6005, Zymo Reseach, Irvine, CA, USA) according to the manufacturer's protocol. The concentration of extracted microorganism DNA was determined using the NanoDrop 1000 Spectrophotometer (Thermo Fisher Scientific, Waltham, MA), and the quality of the DNA was checked by gel electrophoresis (1.5% agarose gel).

Primers 27F (5′‐AGAGTTTGATCCTGGCTCAG‐3′) and 1492R (5′‐GGTTACCTTGTTACGACTT‐3′) were used to amplify the 16S ribosomal RNA (16S rRNA) gene. Additionally, primers P733 (5′‐ATCGAAACGCCTGAAGGTCCAAACAT‐3′) and P734 (5′‐ACACCCTTGTTACCGTGACGACC‐3′) were used to amplify β subunit of bacterial RNA polymerase (RpoB) gene sequence. PCR was performed using Phusion High‐Fidelity PCR Master Mix with HF Buffer (Cat No. F531L, Thermo Fisher Scientific, Waltham, MA, USA), according to the manufacturer's protocol. The reaction conditions were as follows: 30 s denaturation at 98°C, 30 cycles at 98°C for 10 s, annealing for 30 s, and 72°C for 45 s, followed by a final 72°C extension for 5 min. Annealing temperatures were selected based on the primers used: 57.7°C for the 16S rRNA gene and 69.4°C for the RpoB RNA polymerase gene. The amplified PCR products were purified enzymatically using FastAP™ (Cat No. EF0654, Thermo Fisher Scientific, Waltham, MA, USA) and Exonuclease I (Cat No. EN0581, Thermo Fisher Scientific, Waltham, MA, USA) according to manufacturer's protocol. Purified PCR products were sequenced by GENEWIZ from Azenta Life Sciences (Leipzig, Germany). Microorganisms were identified by analyzing sequencing results using Benchling (https://www.benchling.com; accessed on November 1, 2024) and aligning them using NCBI BLAST (National Center for Biotechnology Information, Bethesda, MD, USA; https://blast.ncbi.nlm.nih.gov/Blast.cgi; accessed on December 1, 2024). Accession numbers: Sequences reported in this study were submitted to GenBank with accession numbers for bacterial 16S rRNA PQ867123‐PQ86713 and Rpo gene sequences PQ873103‐PQ873105.

## 3 Results

### 3.1 Sample Collection and Microorganism Cultivation

Forty samples were collected from six caves and an abandoned railway tunnel, now a key roost for a large *Myotis myotis* maternity colony (Table 1). Cave samples were taken from the entrance, twilight, transition, and dark zones, including soil, sediments, moonmilk, mineral deposits, bedrock deposits, insect remains, entomophagous fungi, cave wall biofilm, and guano from various bat species (please refer to Supporting Information S1: Table 1 for more detailed information).

The cultivation of microorganisms from the collected samples was performed by washing a piece of the sample or diluting it with a 0.9% (v/w) NaCl solution, streaking it on the cultivation agar media, and incubating plates until colonies formed, up to 168 h. Two cultivation media types were selected for this study: modified Mueller‐Hinton (mMH), which is more suited for bacterial cultivation; and yeast extract‐peptone‐glycerol (YEPG) for yeast and fungal cultivation. Each sample was incubated on two mMH and YEPG plates at 20°C and 30°C or 37°C temperatures, making a total of 160 plates. The cultivation was considered unsuccessful if no microbial growth was observed after 168 h. The incubation results showed that in all 40 samples, at least one of the four plates had microbial growth. Nevertheless, after 168 h of incubation, 20 mMH and 36 YEPG plates remained empty. The number of colonies exhibiting a distinct morphology (color, texture, size, shape, pigmentation) per plate was as follows: 36 plates contained colonies with only one morphology; 41 plates—colonies with two morphologies; 20 plates—three morphologies; 6 plates—four morphologies; 1 plate—five morphologies. The total number of selected colonies was 207. Supporting Information S1: Table 2 contains more detailed data on individual plates, including those without growth.

### 3.2 Functional Screening on Laccase Activity

**Figure 2:**
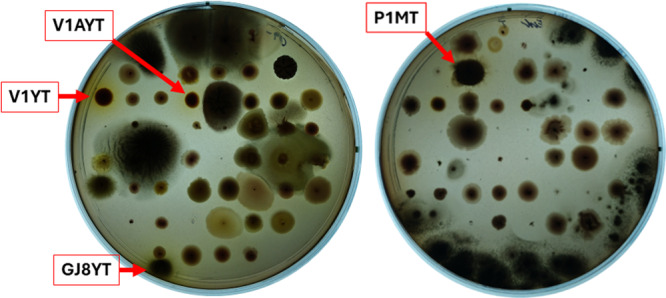
Laccase screening results after 96 h incubation. Left plate—with YEPG‐Ferbamine growth medium; right plate—mMH‐Ferbamine growth medium.

The screening process for laccase activity was incorporated using a cultivation medium augmented with Ferbamine, a substrate designed to detect laccase activity. This substrate undergoes a color change from purple to brown‐yellow upon oxidation, catalyzed by a laccase. Each microorganism culture that exhibited a distinctive morphology on its cultivation plate was transferred onto an appropriate screening plate and then subjected to incubation at the same temperature employed for its cultivation, as described in Section 2. Notably, 29 out of 207 transferred colonies did not demonstrate growth on media with added Ferbamine. The most significant observation of potential laccase activity was recorded after 96 h (Supporting Information S1: Table 3), with three candidates for laccase activity identified on the YEPG‐Ferbamine plate at 30°C and one candidate on the mMH‐Ferbamine plate at 37°C (Figure 2).

The candidate colonies were labeled with their sample code and cultivation plate—YT for YEPG at 30°C and MT for mMH at 37°C. These colonies were then transferred and further grown in larger quantities on appropriate media with Ferbamine to confirm the laccase activity. The results indicated that the GJ8YT growth was not affected by Ferbamine, but the microorganisms showed only minimal laccase activity. Furthermore, colonies V1AYT and V1YT also remained unaffected by Ferbamine, but they specifically formed areas of other types of yellow‐colored compounds than Ferbamine oxidation products. In the initial screening phase on plates, this change was foreseen as a possible presence of laccase activity. The microorganisms from P1MT took 120 h of cultivation to reach a noticeable size and did not indicate possible laccase activity. In short, only one out of four candidate colonies displayed a convincing indication of laccase activity.

The isolation of laccase‐producing microorganisms from GJ8YT was carried out using the streak plate method on mMH, potato dextrose agar (PDA), Luria‐Bertani (LB), Czapek, and YEPG, all supplemented with Ferbamine. After 168 h of incubation, colonies on mMH‐Ferbamine showed the most significant laccase activity (Supporting Information S1: Figure 1). The identification by 16S rRNA region sequencing was performed for all isolated microorganisms from the GJ8YT.

### 3.3 Screening for PU‐Degrading Microorganisms

**Figure 3:**
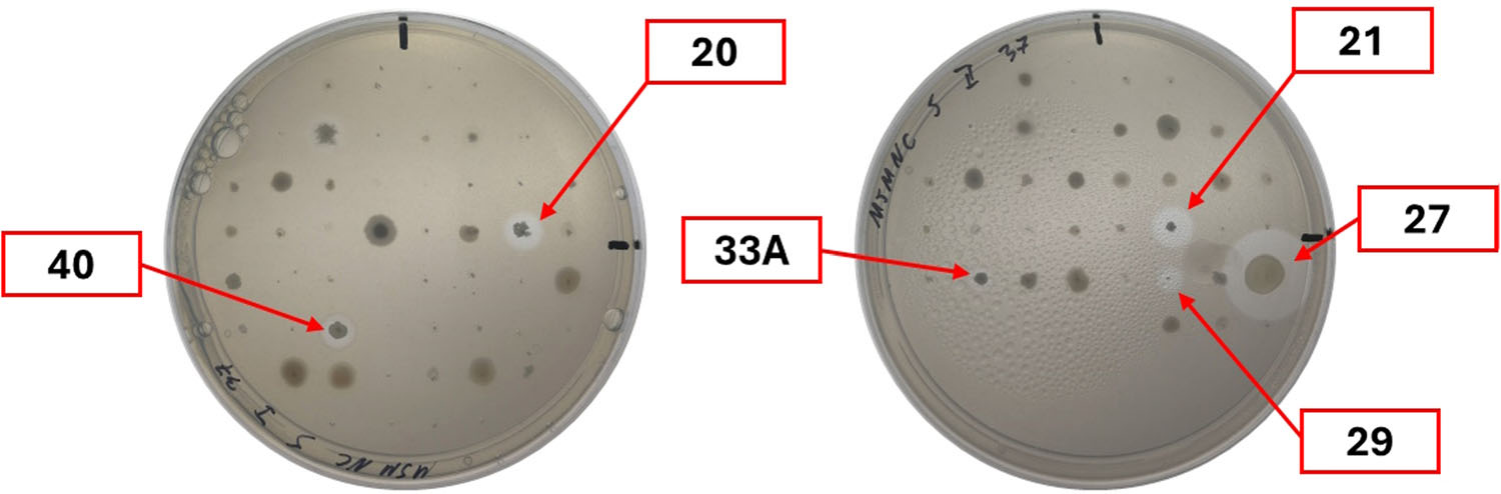
Results for screening for PU degradation after 168 h incubation on MSM + N + C‐PS media at 37°C. PU‐degrading microorganisms are assigned an Isolate ID.

**Figure 4:**
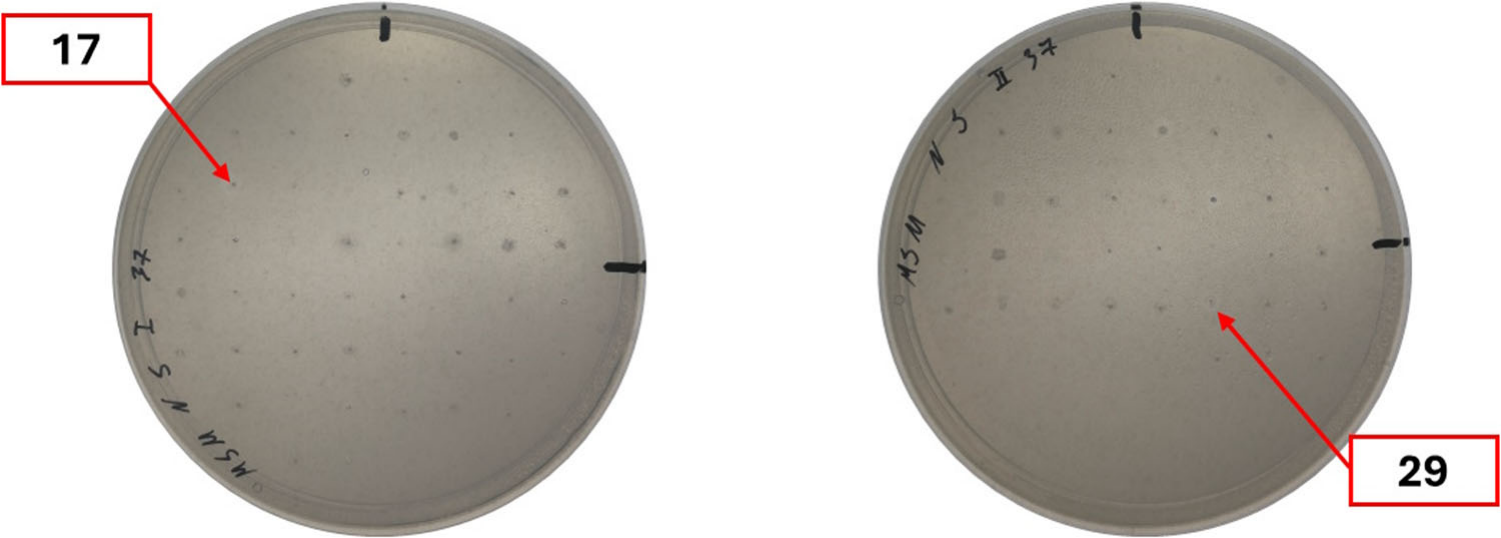
Results for screening for PU degradation after 168 h incubation on MSM + N‐PS media at 37°C. PU‐degrading microorganisms are assigned an Isolate ID.

A polyurethane degradation assay was performed using a Minimal Salt Media (MSM) agar cultivation media supplemented with polyurethane (PU). The presence of colloidal PU particles gives the media a milky appearance, and if PU particle degradation occurs, clear zones form around the microorganisms. Each microorganism culture displaying a distinctive morphology on its cultivation plate was transferred onto every MSM‐PU variant, as described in the Methods section. The screening process was conducted for a duration of up to 168 h, with observations being made at 24‐h intervals. The screening process revealed seven microorganisms capable of degrading polyurethane (PU). Of these seven microorganisms, six exhibited polyester PU degradation activity on MSM + N + C‐PS media (Figure 3), while two exhibited this activity on MSM + N‐PS media (Figure 4). Isolate N° 29 was the only microorganism that exhibited PU‐degrading activity in both media. This indicates that an initial carbon source is unnecessary for its PU degradation and has no inhibitory effect, unlike for isolate N° 17. The readily available carbon and nitrogen sources were essential for promoting initial growth and PU degradation for the other six microorganisms.

No PU degrading activity was detected on MSM media variants supplemented with polyether PU, on MSM media with polyester PU lacking any additional carbon and nitrogen sources, and from all the microorganisms grown at 30°C and 20°C. More detailed screening results are presented in Supporting Information S1: Tables 4 and 5.

### 3.4 Screening for Protease Activity

**Figure 5:**
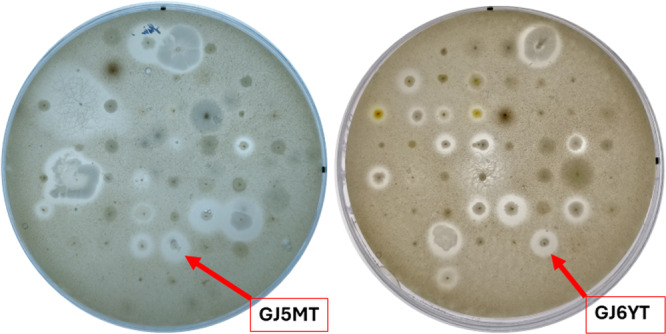
Protease screening results after 24 h of incubation. Left plate—SM plate incubated at 37°C, right plate—SM plate incubated at 30°C temperature.

The protease activity screening was conducted in a manner consistent with the urethanase and laccase activity screenings. In summary, each microorganism culture exhibiting a distinct morphology on its cultivation plate was transferred onto a skim‐milk (SM) agar plate and then incubated on the screening plates at their appropriate cultivation temperatures: 20°C, 30°C, or 37°C. The presence of casein protein micelles in skim milk powder results in the media acquiring a white opacity. In the presence of proteases, these micelles and proteins are degraded, creating clear zones around the microorganisms. The screening results revealed that after just 24 h of incubation, several colonies already showed clear zones: 31 out of 88 colonies at 20°C, 19 out of 54 at 30°C, and 20 out of 65 at 37°C. Representative images of the SM screening plates are presented in Figure 5, with all plates documented in Supporting Information S1: Table 6.

Longer incubation for more than 24 h further widened clearing zones and caused overlap between the colonies, rendering plates unsuitable for analysis. Due to the high prevalence of protease activity, we selected only the GJ5MT and GJ6YT colonies for further investigation, which displayed relatively large clearing zones compared to colony size at 24 h. These colonies were appropriately transferred onto mMH and YEPG plates, cultivated and sequenced for identification.

### 3.5 Identification of Microorganisms

All isolated microorganisms from colonies exhibiting laccase‐like (four strains) and PU biodegradation (seven strains) activities were identified by sequencing their amplified RpoB gene and/or 16S rRNA gene. Two strains with high protease activities were also included in the analysis. The 16S rRNA gene was successfully amplified from all 13 bacteria of interest, whereas the RpoB RNA polymerase gene was amplified from only three. Sequencing generated more than 1390 bp sequences for all 16S rRNA gene samples and variable RpoB RNA polymerase gene sample lengths ranging from 1152 to 1459 bp. Sequences were aligned using NCBI BLAST based on databases—Nucleotide collection (nr/nt) and rRNA/ITS (16S ribosomal RNA sequences), employing megablast for highly similar sequences. The amplification and sequencing results are summarized in Table 2.

**Table 2:** Identified selected bacterial strains based on 16S rRNA and RpoB gene sequence.

Isolate ID	Plate ID	Biocatalytic activity	16S rRNA gene sequence length, bp	Highest homology according to 16S rRNR gene sequence			Highest homology according to RpoB sequence	
				nr/nt database*	rRNA_typestrains/16S_ribosomal_RNA database*	Accession number	nr/nt database*	Accession number
CZA F	GJ8YT	Laccase	1420	*Bacillus* sp. (100%)	*Bacillus* sp. (100%)	PQ867124	*Bacillus subtilis* MB8_B10 CP045824.1 (100%)	PQ873104
PDA F	GJ8YT		1386	*Pseudomonas chlororaphis* subsp. *piscium* DSM 21509, CP027707.1 (100%)	*Pseudomonas chlororaphis* subsp. *aurantiaca* NCIB 10068 NR_164626.1 (99.93%)	PQ867125		
MHB F	GJ8YT		1395	*Pseudomonas chlororaphis* subsp. *piscium* DSM 21509, CP027707.1 (100%)	*Pseudomonas chlororaphis* subsp. *aurantiaca* NCIB 10068 NR_164626.1 (99.93%)	PQ867126		
Y F	GJ8YT		1399	*Pseudomonas chlororaphis* subsp. *piscium* DSM 21509, CP027707.1 (100%)	*Pseudomonas chlororaphis* subsp. *aurantiaca* NCIB 10068 NR_164626.1 (99.93%)	PQ867127		
GJ5MT	GJ5MT	Protease	1398	*Bacillus* sp. (99.79%)	*Bacillus* sp. (99.79%)	PQ867123	*Bacillus subtilis* MB8_B10, CP045824.1 (100%)	PQ873105
GJ6YT	GJ6YT		1405	*Priestia* sp. (100%)	*Priestia* sp. (99.93%)	PQ867128		
17	GJ11MT	PU degradation	1391	*Serratia* sp. (99.86%)	*Serratia liquefaciens* ATCC 27592 NR_122057.1 (99.71%)	PQ867129	*Serratia liquefaciens S1* (99.93%)	PQ873103
20	GJ13MT		1425	*Bacillus stercoris* D7XPN1 NR_181952.1 (99.93%)	*Bacillus stercoris* D7XPN1 NR_181952.1 (99.93%)	PQ867130		
21	PK2MT		1388	*Bacillus* sp. (100%)	*Bacillus pumilus* ATCC 7061 NR_043242.1 (100%)	PQ867131		
27	PK4MT		1411	*Bacillus* sp. (100%)	*Bacillus pumilus* NBRC 12092 NR_112637.1 (100%)	PQ867132		
33 A	PK7MT		1416	*Bacillus* sp. (100%)	*Bacillus pumilus* NBRC 12092 NR_112637.1 (100%)	PQ867133		
40	GS1MT		1404	*Bacillus* sp. (100%)	*Bacillus safensis* NBRC 100820 NR_113945.1 (100%)	PQ867134		
29	PK4MT		1398	*Paenibacillus* sp. (99.86%)	*Paenibacillus mobilis* S8 NR_163642.1 (99.79%)	PQ867135		

* If multiple BLAST results had the same Query Cover and Pairwise identity at the species level with the sequence of interest, only the first strain listed in the results was included in the table. Additionally, if more than one species in the BLAST results had the same Query Cover and Pairwise identity, the sequence of interest was classified solely at the genus level. The values of Query Cover and Pairwise identity are indicated in brackets. Strain accession numbers are indicated after the strain.

Of 13 bacteria of interest, seven belonged to the genus *Bacillus*, three to the genus *Pseudomonas*, and one to *Serratia, Paenibacillus*, and *Priestia*. Multiple species were listed in the NCBI Nucleotide collection (nr/nt) for nine sequences of interest; thus, they were classified solely at the genus level. Sequences of interest were also compared to data in the rRNA type strains/16S rRNA database. Identification results were consistent between the databases and between 16S rRNA and RpoB sequence analysis. Additionally, phylogenetic analysis of the isolated bacterium was done by clustering 16S rRNA gene sequences. Sequences were aligned using (Katoh et al. 2019) version 7 (https://mafft.cbrc.jp/alignment/server/index.html; accessed on April 1, 2025). The tree was constructed using the Neighbor‐joining phylogenetic analysis method and Jukes‐Cantor Substitution model, with a Bootstrap value of 100 replicates. Dendrogram (Supporting Information S1: Figure 2) revealed high bootstrap values, clustering PU degrading microorganisms (N° 27, N° 33 A, N° 21, N° 40) and Ferbamine‐positive (PDA F, MHB F, Y F) in two separate clades.

## 4 Discussion

### 4.1 Sample Collection, Microorganism Cultivation, and Identification

Sampling across six Dinaric karst caves and one abandoned tunnel yielded diverse substrates, including moonmilk, biofilms, guano, and feces. Moonmilk was of particular interest as it has been intensively investigated worldwide for its microbial diversity (Portillo and Gonzalez 2011; Maciejewska et al. 2017; Park et al. 2020). Modified Mueller‐Hinton (mMH) medium was more effective than YEPG for bacterial cultivation, and higher incubation temperatures (20°C–37°C) promoted growth despite the naturally cooler cave climate. These findings suggest that medium composition may be more critical than incubation temperature for recovering cave microorganisms (Bender et al. 2020).

Molecular identification based on the 16S rRNA gene (Woese et al. 1985; Böttger 1989) generally resolved isolates to the genus level, though species‐level assignment was often inconclusive. Nine of thirteen isolates matched multiple taxa in the nr/nt database, while three showed ambiguous type‐strain matches. Supporting RpoB sequencing confirmed results for *Bacillus* and *Serratia* isolates (Singh et al. 2020, 202). Such challenges reflect the presence of multiple rRNA operons and intra‐genomic sequence variation, particularly within the *Bacillus cereus* group (Acinas et al. 2004; Pei et al. 2010; Stoddard et al. 2015; Hakovirta et al. 2016). For instance, isolate 20 A was most similar to *Bacillus stercoris* with a single nucleotide difference, which may represent intra‐genomic polymorphism rather than species divergence (Stoddard et al. 2015). These limitations emphasize the need for complementary genetic markers and highlight the uniqueness of cave‐derived isolates (Oliveira et al. 2017; Turrini et al. 2020; Hathaway et al. 2021; Haidău et al. 2022).

### 4.2 Functional Screening

Among 207 isolates, only one (*Pseudomonas chlororaphis*, GJ8YT) showed convincing laccase‐like activity. This species is known to produce phenazine‐type antibiotics (Chin‐A‐Woeng et al. 2000), and its weak laccase signal may be linked to secondary metabolism (Mandic et al. 2019). The scarcity of laccase‐positive bacteria in our study agrees with previous work suggesting that bacterial laccases are rare in caves (Claus 2004, 200; Santhanam et al. 2011; Kaur et al. 2022), while fungal laccases are more common (Fernández‐Remacha et al. 2022).

By contrast, seven isolates degraded polyester polyurethane (PU), representing, to our knowledge, the first such report from Dinaric karst caves. No isolates degraded polyether PU, in line with studies showing polyester PU is more susceptible to microbial attack (Jin et al. 2022). Enzymatic activity is likely mediated by extracellular esterases (Jiang et al. 2016; Wang et al. 2017). Similar activities have been reported for *Serratia*, *Bacillus*, and *Paenibacillus* (Kim et al. 2022; Ji et al. 2024). One isolate (No. 29) degraded PU without added nutrients, suggesting notable metabolic flexibility.

Protease activity was frequent: 30%–33% of isolates produced clearing zones on skim‐milk agar, exceeding rates reported for soil and plant waste isolates (Nair and Subathra Devi 2024) and comparable to cave‐derived bacteria from the Amazon (Lima and Lobato 2023). Positive strains originated from diverse substrates (soil, biofilm, feces, cocoon, wood), with no clear correlation to sample type or site. This prevalence indicates that extracellular protease production is a widespread adaptive trait in nutrient‐limited cave systems. However, the skim‐milk assay may favor casein‐degrading proteases (Jassim et al. 2024), suggesting additional assays would provide a fuller picture.

### 4.3 Possible Application in the Protection of Subterranean Habitats and Biodiversity Fauna of Dinaric Karst

Microbial communities inhabiting subterranean ecosystems often survive by modifying their metabolic pathways (Rangseekaew and Pathom‐aree 2019; Kosznik‐Kwaśnicka et al. 2022), which undoubtedly affects other cave dwellers and the entire ecosystem within the cave. Understanding their survival strategies can significantly contribute to the discovery of new bioactive molecules, such as enzymes with wide application in biotechnology and medicine, but also facilitate the development of environmentally friendly techniques for removing pollutants and/or toxic substances from the contaminated substrates. Investigating biocatalytic activities in microorganisms has provided an intriguing perspective on subterranean environments. In particular, laccases‐based biosensors have significant practical applications across various fields, including the food industry (Rodríguez‐Delgado et al. 2015), as well as environmental and medical sciences, such as for detecting markers of various pathological conditions (Zhang et al. 2018). Their use in detecting harmful and/or toxic chemicals, including pesticides and phenolic compounds, is crucial in monitoring sensitive habitats such as subterranean and the fauna that inhabit them. For example, bats are a crucial group of mammals, both for their role in ecosystem services and their conservation status. They help regulate insect populations, including pest species in agricultural, forest, and urban environments. Insectivorous bats, particularly, are highly vulnerable to pesticides, which can significantly reduce their populations (EFSA Panel on Plant Protection Products and their Residues et al. 2019). Recent quantification of the benefits of their provision of biological pest control has demonstrated their immense importance in the ecosystem (Frank 2024). In this regard, it is necessary to protect their habitats from pollution, which would significantly contribute to monitoring based on biosensors.

Our finding of polyurethane‐degrading microorganisms from subterranean ecosystems at Ponor Kovači is particularly important because microplastic contamination is becoming a major concern worldwide, including the underground ecosystems (Piccardo and Bevilacqua 2024). Very recently, Balestra and Bellopede (2024) reported on pollution by microplastics and microfibers in the Ponor Kovači—Izvor Ričina Cave System (Bosnia and Herzegovina), including also a few other cave localities in Grabovica Plateau. Some of them were the same localities where the microbial communities were sampled in our study. Actually, Izvor Ričina (Ričina Spring) is an underground continuation of the Šuica River, which sinks on the Ponor Kovači (Kovači Sinkhole) in the small settlement Kovači, on the southwestern edge of the Duvanjsko polje. The entrance to Ponor Kovači and the entrance to Izvor Ričina are approximately 5 km apart, separated by the Grabovica Mountain. According to the latest draft, the total length of the cave system is 3342 m (Rnjak et al. 2021). During the previous expedition, exceptional pollution of aquatic subterranean habitats was already noticed, which also explains the relatively poor cave fauna and decreasing fish populations (Ozimec 2015). It is known that karst aquifers have specific hydrogeological characteristics that make them highly vulnerable to pollution from human activities, as groundwater in karst systems can become contaminated more quickly than in non‐karstic aquifers (Kačaroğlu 1999). Therefore, monitoring microplastic and microfiber pollution in karst areas should be prioritized to protect habitats, conserve species, and manage water resources. Furthermore, one direction for future studies should focus on the potential of bioremediation by *in situ* biostimulation, with an emphasis on indigenous microbial degraders. To optimize this approach and develop environmentally sustainable solutions, further investigation is needed on culturable microorganisms, as well as data on the overall microbial diversity of threatened subsurface habitats. Such efforts could significantly contribute to the protection of subsurface ecosystems and the preservation of their exceptional biodiversity in the Dinaric Karst.

## 5 Conclusion

This study investigated the diversity of cultivable bacteria and enzymatic potential of samples collected from six Dinaric karst caves and an abandoned tunnel, focusing on functional screening for laccase, protease, and polyurethane‐degrading activities. The findings point to the need for further research to identify the microbial diversity of subterranean environments, emphasizing their potential for biotechnological applications and environmental monitoring. Identifying a limited number of microorganisms with laccase and polyurethane‐degrading activities emphasizes the need to further explore subterranean ecosystems as a source of novel enzymes and bioactive molecules. The discovery of polyurethane‐degrading microorganisms is particularly significant given rising concerns over microplastic pollution, including in karst ecosystems. These findings emphasize the importance of protecting vulnerable subterranean habitats and leveraging microbial communities for bioremediation efforts. Additionally, the proteolytic activity of these isolates suggests potential superiority over existing proteases, as their adaptation to the cave's unique chemical and physical conditions may enhance stability and efficiency for industrial applications. The cultivation temperatures (20°C–37°C) were selected to support the growth of mesophilic and moderately thermotolerant microorganisms, which predominate in these subterranean ecosystems. Although the caves maintain lower temperatures (~10°C–15°C), slightly higher temperatures facilitated enzyme detection and are relevant for potential biotechnological applications. Moreover, this study reinforces the importance of understanding subterranean microbial diversity to address pressing environmental challenges, protect sensitive ecosystems, and conserve biodiversity. Further research holds great promise for advancing sustainable solutions for subterranean habitat conservation and pollution management.

## Author Contributions

**Justinas Babinskas:** investigation, writing – original draft, data curation, methodology, visualization. **Jokūbas Krutkevičius:** methodology, visualization, writing – original draft, data curation. **Lada Lukić Bilela:** resources, writing – original draft, data curation, funding acquisition, conceptualization, supervision. **Renata Bešta‐Gajević:** Investigation. **Inga Matijošytė:** project administration, resources, supervision, writing – review and editing, conceptualization, funding acquisition.

## Ethics Statement

The authors have nothing to report.

## Conflicts of Interest

The authors declare no conflicts of interest.

## Supporting information

Supporting Material Revised.

## Data Availability

The data that support the findings of this study are available from the corresponding author upon reasonable request. The authors declare that the data supporting the findings of this study are available within the paper and its Supporting Information files. Should any raw data files be needed in another format they are available from the corresponding author upon reasonable request.
