# Supplementary material for: Potential of Subterranean Microbes: High‐Throughput Screening for Industrially Relevant Enzymatic Activities in Dinaric Caves
Source: Microbiologyopen. 2025 Nov 5;14(6):e70146. doi: 10.1002/mbo3.70146 (PMC12589811; doi:10.1002/mbo3.70146)
Supplement: Supplementary file 1 — Supporting Material Revised. [file MBO3-14-e70146-s001.docx]

**Supplementary Material**

**Supplementary Figure 1**. Results of laccase activity in microorganisms isolated from GJ8YT, after incubation on mHM-Ferbamine agar for 198 h at 37 °C.


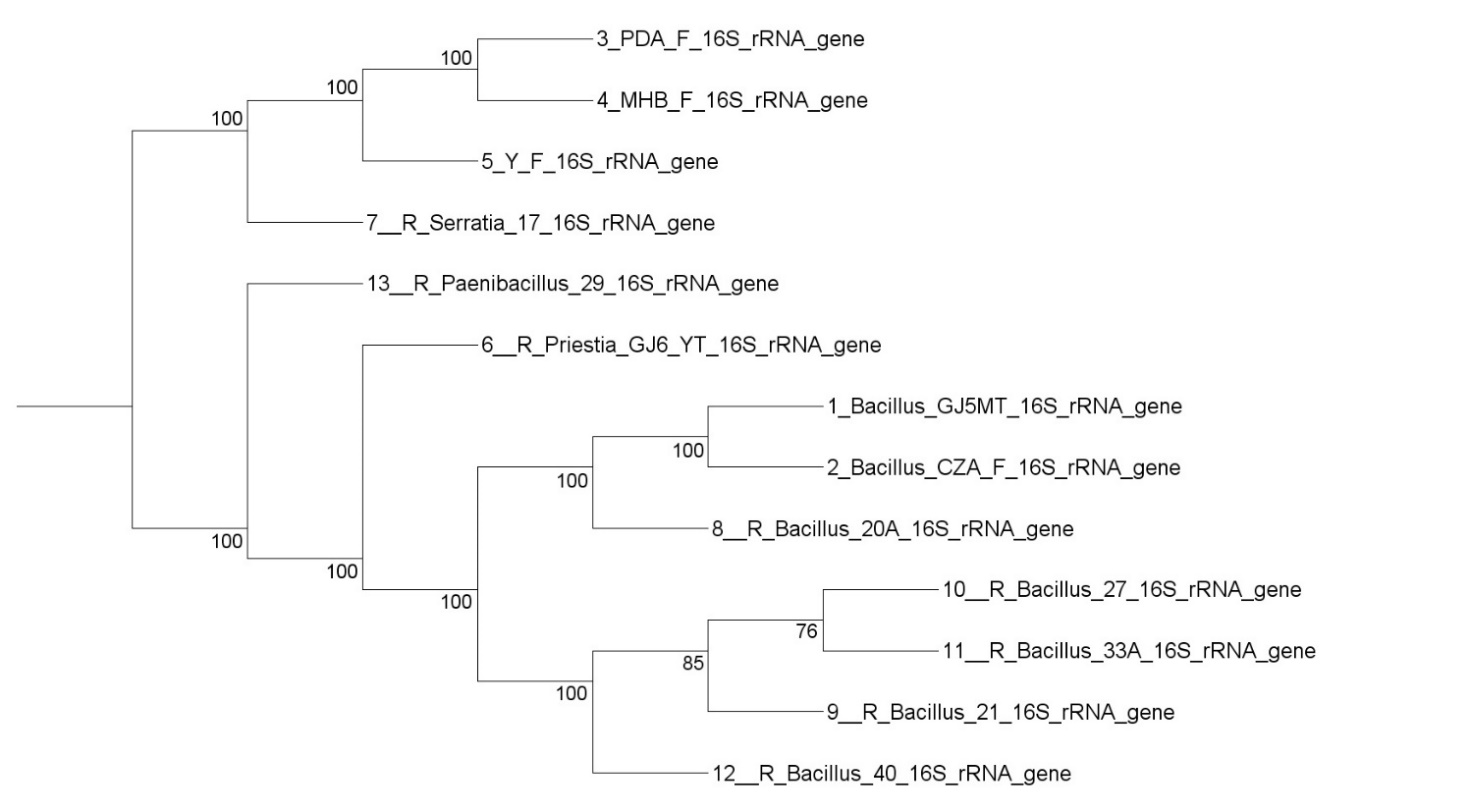


**Supplementary Figure 2.** Phylogenetic analysis of the isolated bacterium by clustering 16S rRNA gene sequences.

**Supplementary Table 1.** The sample types with the codes.

| **Gornja Jakovljeva Cave**  **Dinara Mt.** | **Sample type** | **Code** |  |
| --- | --- | --- | --- |
|  | Microbial biofilm | GJ1-2, GJ7, GJ12-16 | **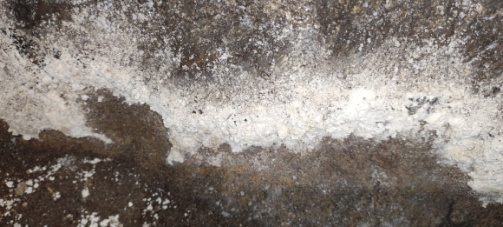** 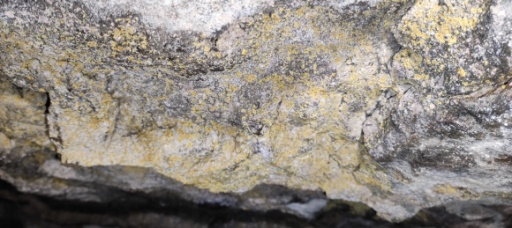  Moonmilk (left) and microbial biofilm (right) on the cave walls (Photo: Lada Lukić Bilela) |
|  | *Unidentified* | GJ3, GJ4 |  |
|  | Sediment | GJ5 |  |
|  | Moonmilk | GJ6, GJ8 |  |
|  | Clay | GJ9 |  |
|  | Fungi on faeces | GJ11 |  |
|  | Wood | GJ17 |  |
| **Donja Mijatova Cave**  **Vran Mt.** | **Sample type** | **Code** | 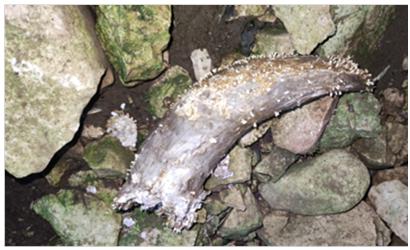 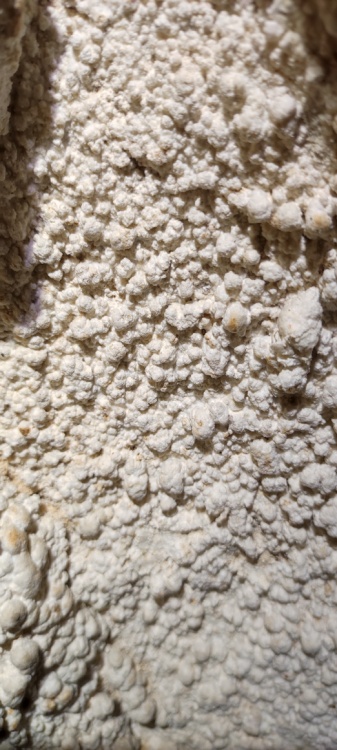 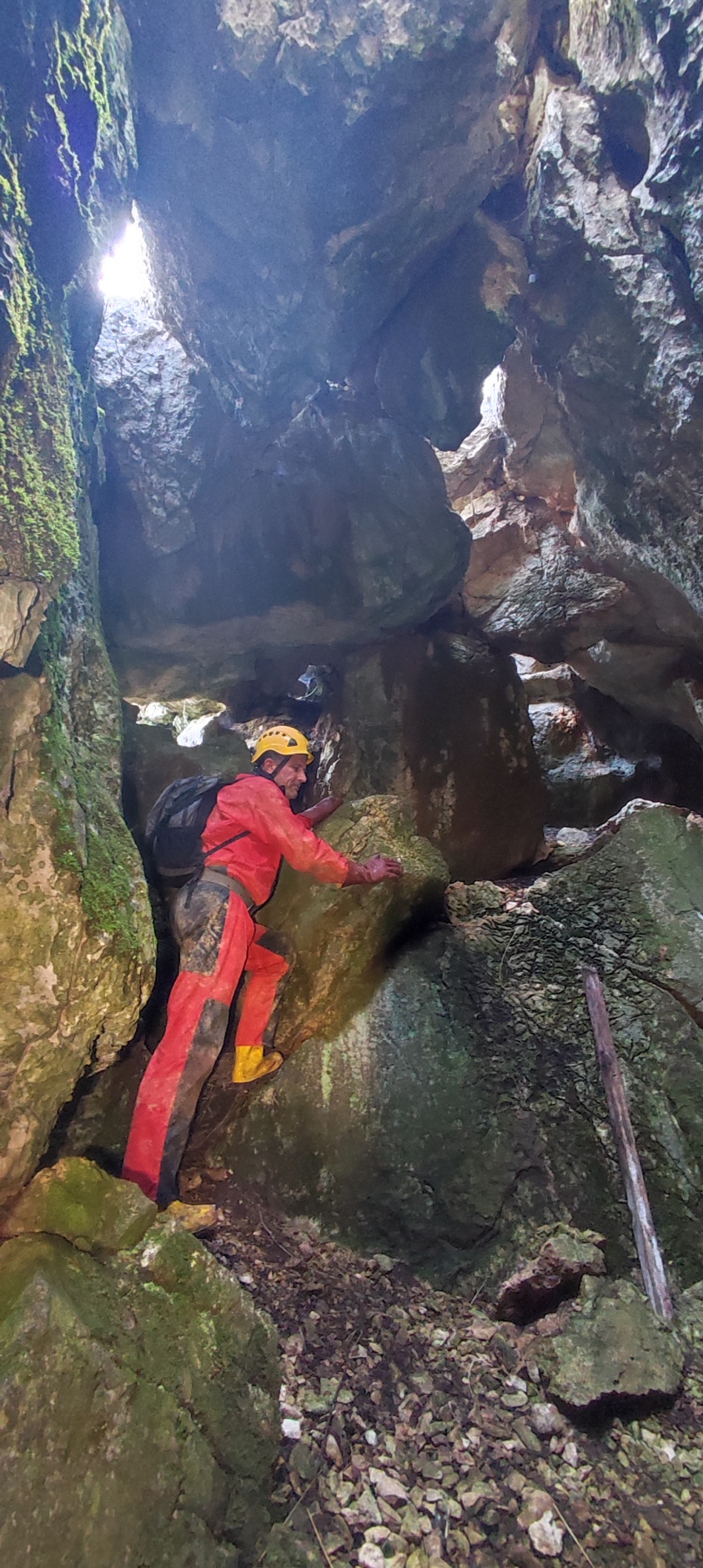  Bovine horn with a horn stalkball *Onygena equina* ((left), cave wall biofilm (middle), wood (right) (Photo: Lada Lukić Bilela) |
|  | Horn with fungi* | DM1 |  |
|  | Fungi | DM2 |  |
|  | Wood | DM3 |  |
|  | Stalagmite | DM4 |  |
| **Ponor Kovači**  **Grabovica Plateau** | **Sample type** | **Code** | 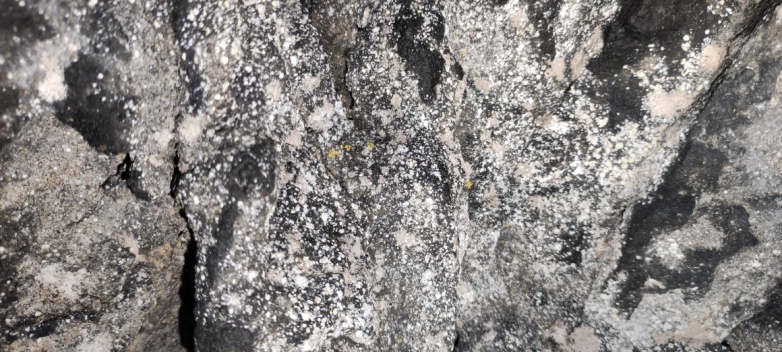  Microbial biofilm on the cave wall (Photo: Lada Lukić Bilela) |
|  | Wood | PK1 |  |
|  | Soil | PK2 |  |
|  | Fungi | PK3 |  |
|  | Biofilm | PK4 |  |
|  | Fungi on insect (*Stenophylax* sp.) | PK5 |  |
|  | Fungi on the Opiliones | PK6 |  |
|  | Faeces (*Martes marte*s) | PK7 |  |
|  | Fungi on roots | PK8 |  |
| **Prosječenica Cave**  **Podveležje** | **Sample type** | **Code** | **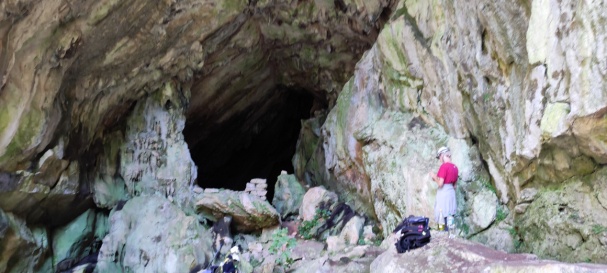** 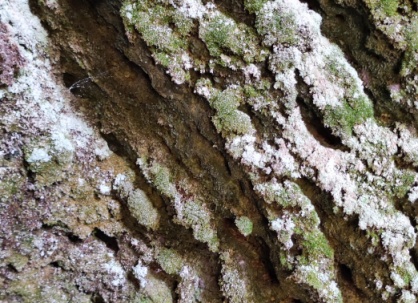  The entrance (left) and bat colony (right) (Photo: Lada Lukić Bilela) |
|  | Bat guano | P1 |  |
|  | Cave soil | P2 |  |
| **Vakuf Cave**  **Gornja Studenčica** | **Sample type** | **Code** | **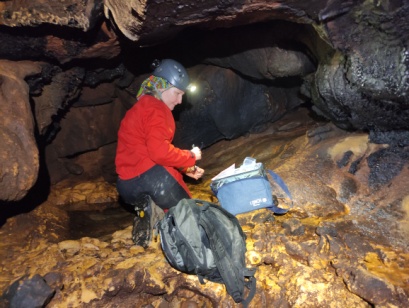** 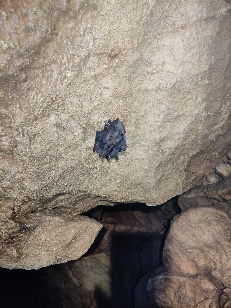  Sampling in the Vakuf Cave (left) and bat colony (right) (Photo: Sašo Finžgar) |
|  | A piece of wood | GS1 |  |
|  | Guano with fungi | GS2 |  |
|  | Deposits on spider net | GS3 |  |
|  | Cocoon with fungi | GS4 |  |
|  | Bat guano | GS5 |  |
| **Čvaljina Tunnel**  **Čvaljina, Popovo polje** | **Sample type** | **Code** | **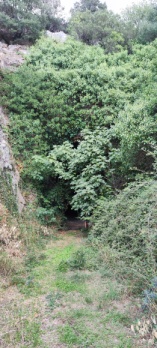**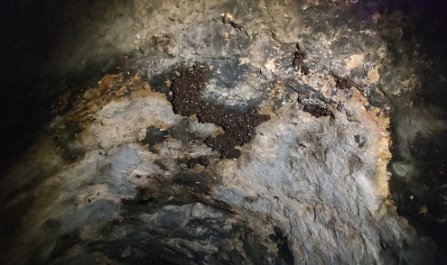  The entrance (left) and bat colony (right) (Photo: Lada Lukić Bilela) |
|  | Dry bat guano | TČ1 |  |
|  | Guano with soil | TČ2 |  |
| **Vjetrenica Cave**  **Zavala, Popovo Polje** | **Sample type** | **Code** | **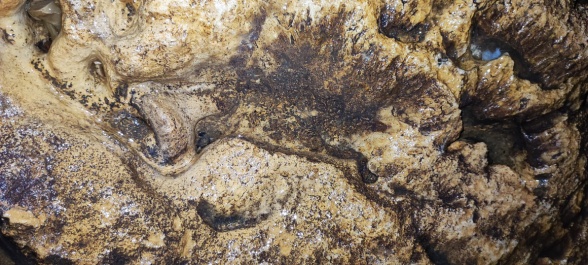**  Sediment from the Donje Jezero (The Lower Lake) (Photo: Lada Lukić Bilela) |
|  | Soil and sediment | V1A |  |
|  | Lake sediment | V1 |  |
|  | *Unidentified* | V2 |  |

**Supplementary Table 2**. Results of microorganism cultivation and functional screening.

| Microorganism cultivation | | | | Functional analysis*^c^* | | | |  |
| --- | --- | --- | --- | --- | --- | --- | --- | --- |
| Sample code*^a^* | Sample  type | Cultivation conditions*^b^* | Morphologies | Laccase, 96 h | Urethanase (ester), 96 h | Urethenase (ether), 96 h | Protease, 24 h | |
| GS1 | Wood | MT | 2 |  | **+** |  | **++** | |
|  |  | M | 3 |  |  |  |  | |
|  |  | YT | 3 |  |  |  | **++** | |
|  |  | Y | 4 |  |  |  | **+** | |
| GS2 | Guano with fungi | MT | 0 |  |  |  |  | |
|  |  | M | 2 |  |  |  |  | |
|  |  | YT | 2 |  |  |  |  | |
|  |  | Y | 0 |  |  |  |  | |
| GS3 | Deposits on spider net | MT | 3 |  |  |  | **+++** | |
|  |  | M | 1 |  |  |  |  | |
|  |  | YT | 4 |  |  |  | **+** | |
|  |  | Y | 0 |  |  |  |  | |
| GS4 | Cocoon with fungi | MT | 1 |  |  |  |  | |
|  |  | M | 1 |  |  |  | **+** | |
|  |  | YT | 2 |  |  |  | **++** | |
|  |  | Y | 3 |  |  |  | **+/+++** | |
| GS5 | Bat guano | MT | 2 |  |  |  | **+** | |
|  |  | M | 2 |  |  |  | **+++** | |
|  |  | YT | 2 |  |  |  |  | |
|  |  | Y | 0 |  |  |  |  | |
| P1 | Bat guano | MT | 1 | + (false positive) |  |  |  | |
|  |  | M | 1 |  |  |  |  | |
|  |  | YT | 1 |  |  |  |  | |
|  |  | Y | 0 |  |  |  |  | |
| P2 | Soil | MT | 3 |  |  |  | **+** | |
|  |  | M | 2 |  |  |  | **+** | |
|  |  | YT | 0 |  |  |  |  | |
|  |  | Y | 2 |  |  |  |  | |
| TČ1 | Dry bat guano | MT | 1 |  |  |  | **+** | |
|  |  | M | 2 |  |  |  | **+++** | |
|  |  | YT | 0 |  |  |  |  | |
|  |  | Y | 2 |  |  |  |  | |
| TČ2 | Guano with soil | MT | 0 |  |  |  |  | |
|  |  | M | 1 |  |  |  |  | |
|  |  | YT | 0 |  |  |  |  | |
|  |  | Y | 0 |  |  |  |  | |
| V1A | Soil and sediment | MT | 3 |  |  |  | **+/+** | |
|  |  | M | 1 |  |  |  | **+++** | |
|  |  | YT | 3 | + (false positive) |  |  | **+/+/+** | |
|  |  | Y | 1 |  |  |  | **++** | |
| V1 | Lake sediment | MT | 2 |  |  |  | **++** | |
|  |  | M | 0 |  |  |  |  | |
|  |  | YT | 2 | + (false positive) |  |  | **+** | |
|  |  | Y | 0 |  |  |  |  | |
| V2 | *Unidentified* | MT | 0 |  |  |  |  | |
|  |  | M | 0 |  |  |  |  | |
|  |  | YT | 0 |  |  |  |  | |
|  |  | Y | 1 |  |  |  |  | |
| DM1 | Horn with Fungi | MT | 1 |  |  |  | **++** | |
|  |  | M | 1 |  |  |  | **+++** | |
|  |  | YT | 1 |  |  |  | **++** | |
|  |  | Y | 0 |  |  |  |  | |
| DM2 | Fungi | MT | 2 |  |  |  | **++** | |
|  |  | M | 1 |  |  |  | **++** | |
|  |  | YT | 1 |  |  |  |  | |
|  |  | Y | 1 |  |  |  | **+++** | |
| DM3 | Wood | MT | 2 |  |  |  | **+** | |
|  |  | M | 2 |  |  |  | **++** | |
|  |  | YT | 4 |  |  |  | **++/++** | |
|  |  | Y | 3 |  |  |  | **+** | |
| DM4 | Stalagmite | MT | 0 |  |  |  |  | |
|  |  | M | 2 |  |  |  |  | |
|  |  | YT | 2 |  |  |  |  | |
|  |  | Y | 1 |  |  |  | **++** | |
| PK1 | Wood | MT | 1 |  |  |  |  | |
|  |  | M | 2 |  |  |  | **+** | |
|  |  | YT | 3 |  |  |  |  | |
|  |  | Y | 4 |  |  |  |  | |
| PK2 | Soil | MT | 3 |  | **++** |  |  | |
|  |  | M | 0 |  |  |  |  | |
|  |  | YT | 4 |  |  |  | **+/++** | |
|  |  | Y | 1 |  |  |  | **+** | |
| PK3 | Fungi | MT | 2 |  |  |  |  | |
|  |  | M | 0 |  |  |  |  | |
|  |  | YT | 0 |  |  |  |  | |
|  |  | Y | 2 |  |  |  | **+** | |
| PK4 | Biofilm | MT | 3 |  | **+++/+** |  | **++** | |
|  |  | M | 1 |  |  |  | **++** | |
|  |  | YT | 3 |  |  |  | **++** | |
|  |  | Y | 3 |  |  |  | **+/+/++** | |
| PK5 | Fungi on insect | MT | 2 |  |  |  | **++** | |
|  |  | M | 0 |  |  |  |  | |
|  |  | YT | 2 |  |  |  | **+++/++** | |
|  |  | Y | 0 |  |  |  |  | |
| PK6 | Fungi on insect | MT | 1 |  |  |  |  | |
|  |  | M | 0 |  |  |  |  | |
|  |  | YT | 0 |  |  |  |  | |
|  |  | Y | 1 |  |  |  | **++** | |
| PK7 | Faeces (*Martes martes*) | MT | 3 |  | **+** |  | **+++/+++** | |
|  |  | M | 2 |  |  |  | **++/++** | |
|  |  | YT | 0 |  |  |  |  | |
|  |  | Y | 0 |  |  |  |  | |
| PK8 | Fungi on roots | MT | 2 |  |  |  | **+** | |
|  |  | M | 0 |  |  |  |  | |
|  |  | YT | 0 |  |  |  |  | |
|  |  | Y | 2 |  |  |  | **++/+++** | |
| GJ1 | Biofilm | MT | 0 |  |  |  |  | |
|  |  | M | 2 |  |  |  | **+/++** | |
|  |  | YT | 2 |  |  |  |  | |
|  |  | Y | 0 |  |  |  |  | |
| GJ2 | Biofilm | MT | 0 |  |  |  |  | |
|  |  | M | 0 |  |  |  |  | |
|  |  | YT | 1 |  |  |  |  | |
|  |  | Y | 2 |  |  |  |  | |
| GJ3 | *Unidentified* | MT | 2 |  |  |  | **++** | |
|  |  | M | 1 |  |  |  | **++** | |
|  |  | YT | 1 |  |  |  |  | |
|  |  | Y | 0 |  |  |  |  | |
| GJ4 | *Unidentified* | MT | 2 |  |  |  |  | |
|  |  | M | 1 |  |  |  | **+++** | |
|  |  | YT | 0 |  |  |  |  | |
|  |  | Y | 0 |  |  |  |  | |
| GJ5 | Sediment | MT | 2 |  |  |  | **+++/++** | |
|  |  | M | 0 |  |  |  |  | |
|  |  | YT | 0 |  |  |  |  | |
|  |  | Y | 2 |  |  |  |  | |
| GJ6 | Moonmilk | MT | 2 |  |  |  |  | |
|  |  | M | 2 |  |  |  |  | |
|  |  | YT | 3 |  |  |  | **+++** | |
|  |  | Y | 0 |  |  |  |  | |
| GJ7 | Biofilm | MT | 3 |  |  |  |  | |
|  |  | M | 0 |  |  |  |  | |
|  |  | YT | 5 |  |  |  | **++** | |
|  |  | Y | 0 |  |  |  |  | |
| GJ8 | Moonmilk | MT | 1 |  |  |  |  | |
|  |  | M | 1 |  |  |  |  | |
|  |  | YT | 1 | **+** |  |  | **+** | |
|  |  | Y | 1 |  |  |  | **++** | |
| GJ9 | Clay | MT | 0 |  |  |  |  | |
|  |  | M | 3 |  |  |  |  | |
|  |  | YT | 0 |  |  |  |  | |
|  |  | Y | 3 |  |  |  |  | |
| GJ11 | Fungi from faeces | MT | 1 |  | **+** |  | **+** | |
|  |  | M | 1 |  |  |  |  | |
|  |  | YT | 0 |  |  |  |  | |
|  |  | Y | 0 |  |  |  |  | |
| GJ12 | Biofilm | MT | 2 |  |  |  |  | |
|  |  | M | 0 |  |  |  |  | |
|  |  | YT | 0 |  |  |  |  | |
|  |  | Y | 0 |  |  |  |  | |
| GJ13 | Biofilm | MT | 1 |  | **+** |  | **++** | |
|  |  | M | 2 |  |  |  | **++** | |
|  |  | YT | 0 |  |  |  |  | |
|  |  | Y | 0 |  |  |  |  | |
| GJ14 | Biofilm | MT | 4 |  |  |  | **+** | |
|  |  | M | 0 |  |  |  |  | |
|  |  | YT | 0 |  |  |  |  | |
|  |  | Y | 2 |  |  |  | **+** | |
| GJ15 | *Unidentified* | MT | 1 |  |  |  |  | |
|  |  | M | 0 |  |  |  |  | |
|  |  | YT | 0 |  |  |  |  | |
|  |  | Y | 2 |  |  |  | **++** | |
| GJ16 | *Unidentified* | MT | 1 |  |  |  |  | |
|  |  | M | 2 |  |  |  | **++** | |
|  |  | YT | 0 |  |  |  |  | |
|  |  | Y | 0 |  |  |  |  | |
| GJ17 | Wood | MT | 3 |  |  |  | **++** | |
|  |  | M | 3 |  |  |  |  | |
|  |  | YT | 2 |  |  |  | **+++** | |
|  |  | Y | 0 |  |  |  |  | |

*^a^* Sampling site abbreviation: GS – Vakuf cave, P – Prosječeni cave, TČ – Čvaljina tunnel, V – Vjetrenica cave, DM – Donja Mitjatova cave, PK – Ponor Kovači, GJ – Gornja Jakovljeva cave.

*^b^* MT – modified Mueller-Hinton agar at 37 °C; M – modified Mueller-Hinton agar at 20 °C; YT – yeast extract-peptone-glycerol agar at 30 °C; Y – yeast extract-peptone-glycerol agar at 20 °C.

*^c^* Colour legend: black – not screened due to lack of colony growth on the cultivation plate; red – no colony growth on the screening plate; blank – catalytic activity not detected; green – positive enzymatic activity (+++ significant, ++ moderate, + perceptible).

**Supplementary Table 3**. Screening results for laccase activity after 96 h incubation on mHM and YEPG media augmented with Ferbamine.

| Incubation temperatures | mHM + Ferbamine | YEPG + Ferbamine |
| --- | --- | --- |
| 20 °C |  |  |
| 37 °C for mHM  30 °C for YEPG |  |  |

**Supplementary Table 4**. Screening results for PU degradation, after 168 h incubation on MSM media variants, containing polyester (PS) or polyether (PE) PU, at 37 °C.

|  | PS PU | PE PU |
| --- | --- | --- |
| MSM+N+C | 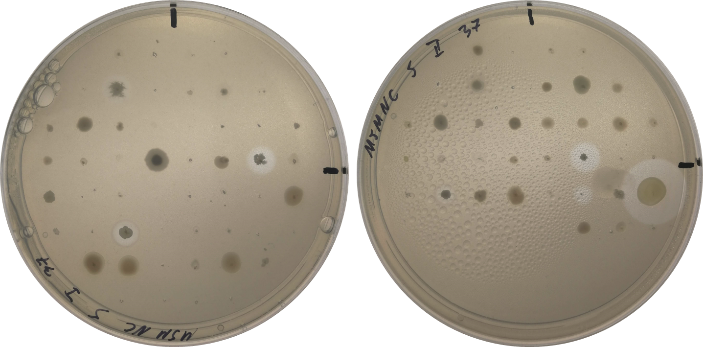 | 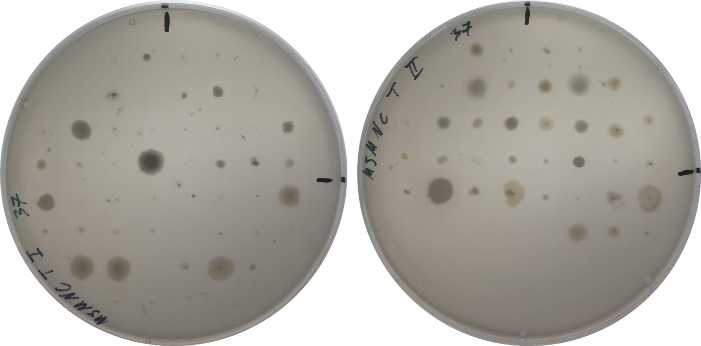 |
| MSM+N | 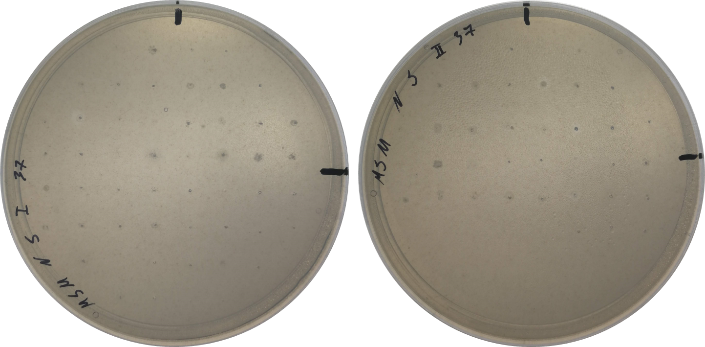 | 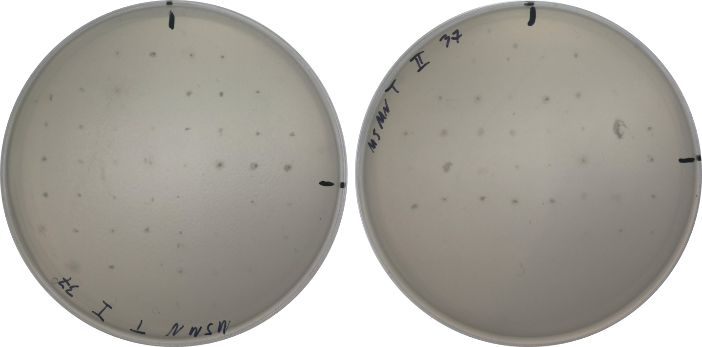 |

**Supplementary Table 5**. Screening results for PU degradation, after 168 h incubation on MSM media variants, containing polyester (PS) or polyether (PE) PU, at 30 °C.

|  | PS PU | PE PU |
| --- | --- | --- |
| MSM+N+C | 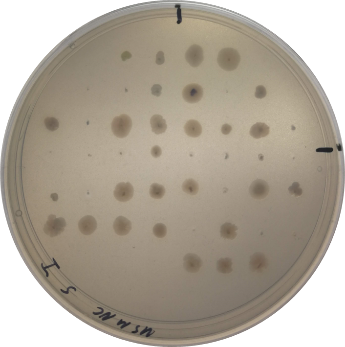 | 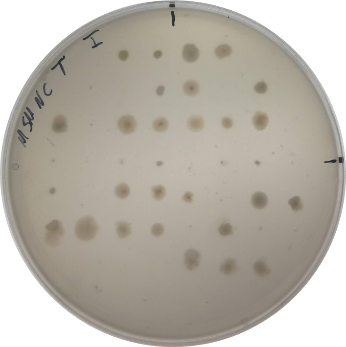 |
| MSM+N | 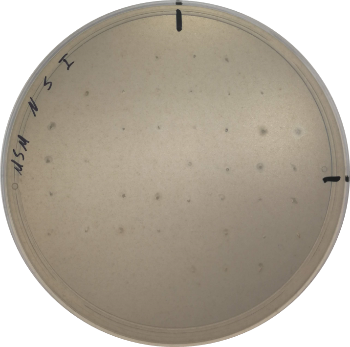 | 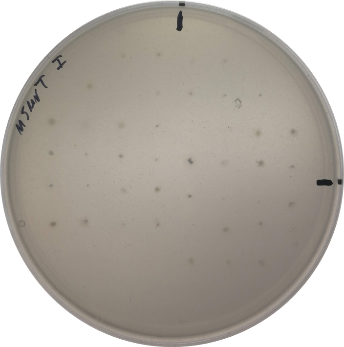 |

**Supplementary Table 6**. Screening results for protease activity after 24 h incubation on skim-milk agar plates at 20 °C, 30 °C and 37 °C.

| 20 °C |  |
| --- | --- |
| 30 °C |  |
| 37 °C |  |
